# A Noise-Tolerating Gene Association Network Uncovering an Oncogenic Regulatory Motif in Lymphoma Transcriptomics

**DOI:** 10.3390/life13061331

**Published:** 2023-06-06

**Authors:** Wei-Quan Fang, Yu-Le Wu, Ming-Jing Hwang

**Affiliations:** 1 Institute of Biomedical Sciences, Academia Sinica, Taipei 115, Taiwan; waltwu512@gmail.com; 2 Division of New Drug, Center for Drug Evaluation, Taipei 115, Taiwan

**Keywords:** cancer prognostic genes, gene association network, diffuse large B-cell lymphoma, biological noises

## Abstract

In cancer genomics research, gene expressions provide clues to gene regulations implicating patients’ risk of survival. Gene expressions, however, fluctuate due to noises arising internally and externally, making their use to infer gene associations, hence regulation mechanisms, problematic. Here, we develop a new regression approach to model gene association networks while considering uncertain biological noises. In a series of simulation experiments accounting for varying levels of biological noises, the new method was shown to be robust and perform better than conventional regression methods, as judged by a number of statistical measures on unbiasedness, consistency and accuracy. Application to infer gene associations in germinal-center B cells led to the discovery of a three-by-two regulatory motif gene expression and a three-gene prognostic signature for diffuse large B-cell lymphoma.

## 1. Introduction

The network modeling of biological systems can capture many of their essential characteristics [1,2]. In such modeling, biological processes are often depicted as a simple network graph where nodes represent molecules and edges that connect nodes represent their interactions or associations [3]. Although seemingly simplistic, mathematical and numerical simulations of prototype biological networks have served to provide insight into unknown structures or relationships of gene associations and regulations (e.g., [4]). A number of methods exploiting different algorithms have been developed to construct gene association networks (GANs), including graphical Gaussian models [5], Bayesian networks [6,7], and models of other approaches (see [8] for a comprehensive review).

### Importance of Modeling Gene Association Networks with Biological Noises

One key issue for most of these GAN construction studies is that they assume gene expressions follow a known and well-defined probability distribution function, often a normal distribution function, i.e., a Gaussian probability function. This assumption may significantly depart from actuality, however, as gene expression is known to be influenced by non-Gaussian stochastic noises [9,10]. How the uncertainties in gene expressions and their noises are handled can have a significant impact on the resultant GAN and hence its predicted biological behaviors. Such uncertainties, called biological noises, can arise from, for example, stochastic oscillations in gene expressions [11], which can, in general, be categorized into either intrinsic or extrinsic [12]. Intrinsic noises may come from various sources, including individual events of transcription and translation, rates of biochemical reaction, or species concentrations [12,13], while extrinsic noises may be induced by external factors such as pathogens and other foreign compounds such as pharmaceuticals and vaccines [14]. Because the combined effects of these noises are often quite complicated to model or analyze, many investigations do not consider them or simply assume that their effects are small and, therefore, can be ignored [15,16], even though models can yield non-significant statistical results [17] or wrong predictions [16] if these inherent complexities are not addressed.

Using differential equations to model GANs is a well-developed approach that can tolerate perturbations of noises [18,19]. However, one limitation of this approach is its requirement of time series data, which excludes direct applications to many useful non-temporal datasets, although special handling can be developed as in the example of an analytical procedure based on steady-state treatments [20]. Additionally, the main challenge of working with differential equations is that there are no closed-form solutions for them [21]. Finally, there is a tendency for the approach of differential equations to incur high computational costs, even for a small network with less than half a dozen nodes [18].

Another approach to model GANs is regression-based. However, most of the regression-based studies either do not account for the effects of intrinsic and extrinsic noises or lump them together and model them using a normal distribution function (see, e.g., [6,7,22]). For example, using a least squares (LS)-based regression approach, an attempt to study the impact of errors or variations arising from measuring processes on the identification of a GAN was presented by Fujita and coworkers [23], in which experimental observations and random errors were necessarily assumed normally distributed for robust parameter estimations. Another pitfall of Fujita et al.’s approach is that biological noises for predictor genes were neglected in their model setup, not to mention that LS-based methods are known to be unstable when data are highly correlated, i.e., ill-conditioned or having multi-collinearity [24].

The aforementioned problems motivated us to use a distribution-free regression method to consider biological uncertainties, i.e., unknown distributions of gene expressions and their noises, in GAN modeling. As we showed below, this method, called AWTE, which is based on a new statistical method of consistent estimation developed by one of us [17], has several advantages. First, it can achieve outstanding statistical properties in handling noises, Gaussian or non-Gaussian. Second, there is no need to solve objective functions in estimating the association parameters, and therefore the computational cost is considerably cheaper than methods that require the use of an optimization algorithm. Third, the method can cope with non-temporal observations and thus is suitable for applications to many expression datasets where time series data are not available. Although all of the regression methods mentioned in this article can, in theory, be distribution-free in dealing with data uncertainties, the conventional methods cannot be directly applied to model a GAN with manifold biological noises in such a manner, and, as the results of our numerical simulations show, they are not robust and would perform poorly when uncertainties are substantial. The robustness of the present method was also shown by its better ability than conventional regression methods to infer a GAN of germinal center B-cell genes from transcriptomes of lymphoma tumors that could reproduce experimental observations.

**Figure A1:**
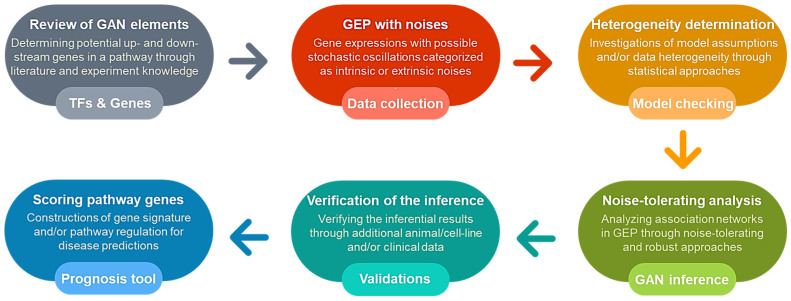
**Flow chart for discovering disease-causing gene regulation motif using noise-tolerating GAN approach**. GEP: Gene Expression Profile.

This study aimed at developing a general bioinformatics process that can determine potential disease-causing gene regulations using a new, noise-tolerating regression-based approach to gene expression data. We illustrated this process in Figure A1 (Appendix B).

## 2. Materials and Methods

### 2.1. A Framework for Regression-Based Modeling of GAN

Let us suppose that the research objective is to figure out how *q* target genes *Y*_1_, *Y*_2_,*…*, *Y_q_* are associated with *p* predictor genes *X*_1_, *X*_2_,*…*, *X_p_*, and *n* independent experiments (e.g., microarrays) are conducted for this purpose, in which for the *i*-th experiment, the observed expression levels of genes *Y_j_* and *X_k_* are *y_ij_* and *x_ik_*, respectively, but for convenience, we will drop the subscript *I* here. A regression model for the GAN of *q* simultaneous equations can then be expressed as [23]:

(1) $$\begin{array}{c} y_{1}^{}=\beta_{11}^{}x_{1}+\beta_{12}^{}x_{2}+\cdots +\beta_{1p}^{}x_{p}+\varepsilon_{y1}^{}\\ y_{2}^{}=\beta_{21}^{}x_{1}+\beta_{22}^{}x_{2}+\cdots +\beta_{2p}^{}x_{p}+\varepsilon_{y2}^{}\\ \;\;\;\;\;\vdots \\ y_{q}^{}=\beta_{q1}^{}x_{1}+\beta_{q2}^{}x_{2}+\cdots +\beta_{\mathrm{qp}}^{}x_{p}+\varepsilon_{\begin{array}{c} yq \end{array}}^{} \end{array}$$

where the error terms, *ε_y_*_1_, *ε_y_*_2_, …, *ε_yq_*, as well as the expression levels of genes *X*s and *Y*s, are random variables (i.e., non-constants).

We used the architecture of (1) for GAN construction mainly for two reasons. First, even though distribution-free modeling under the regression framework has been reported, we would like to develop a new approach with fewer assumptions. Second, unlike other approaches, such as Bayesian models, our approach could use a standard *p*-value cutoff of 0.05 to infer an association under the architecture of (1). This is attractive, especially when prior knowledge concerning the gene’s regulation role in GAN is lacking.

### 2.2. Conventional Strategies for Estimating the Association Parameters

Generally speaking, Equation (1) can be a distribution-free regression model of GAN if we do not specify a probability density function for the expression and error terms, but in previous studies, including the work of [23], a normal distribution function was used to model gene expressions and measuring noises. Note also that although [25] had shown that large errors or outliers of expression data do not need to be modeled by a Gaussian distribution function in regression-based inferring of gene regulatory networks, their method nonetheless required all the errors and outliers to be modeled as symmetrically distributed residuals, which are unrealistic for real-world non-Gaussian noises. For the *i*-th observation (experiment), a regression model of the *j*-th equation of Equation (1) can be rewritten as follows:

(2) $$y_{\mathrm{ij}}^{}=\beta_{j1}^{}x_{i1}+\beta_{j2}^{}x_{i2}+\cdots +\beta_{\mathrm{jp}}^{}x_{\mathrm{ip}}+\varepsilon_{\mathrm{yij}}^{}$$

If we do not know the specific probability density functions for the observed expressions and error terms, there are, in general, three conventional LS-based strategies to estimate the association parameters of *β* in Equation (2). The first one is the ordinary LS estimation (LSE) strategy, for which the association can be detected by minimizing the following objective function Q*_j_*_0_ based on the sum of squares of the error terms *ε_yij_*’s [26]

(3) $$Q_{j0}^{}\left(\beta_{j1}^{},\cdots ,\beta_{\mathrm{jp}}^{}\right)={\sum_{i=1}^{n}\left(y_{\mathrm{ij}}^{}-\sum_{k=1}^{p}\beta_{\mathrm{jk}}^{}x_{\mathrm{ik}}^{}\right)}_{}^{2}$$

Assuming that these error terms are independently and identically distributed with zero mean and finite variance, LSE has some well-behaved statistical properties, including unbiasedness and minimal variance, as summarized by the Gauss–Markov theorem [26].

The second one is the *L*_1_-norm penalized strategy, called least absolute shrinkage and selection operator, or LASSO [27], for which the association of Equation (2) can be obtained by minimizing Q*_j_*_1_, using an *L*_1_ penalty on top of Equation (3)

$$Q_{j1}^{}\left(\lambda_{\;j1}^{},\beta_{j1}^{},\cdots ,\beta_{\mathrm{jp}}^{}\right)=Q_{j0}^{}\left(\beta_{j1}^{},\cdots ,\beta_{\mathrm{jp}}^{}\right)+\lambda_{\;j1}^{}\sum_{\begin{array}{c} k=1 \end{array}}^{p}\left|\beta_{\mathrm{jk}}^{}\right|$$

where *λ_j_*_1_ is the tuning (weight) parameter in the penalty term, *L*_1_, of Q*_j_*_1_.

The third one is the *L*_2_-norm penalized strategy, called ridge regression estimation, or RRE [28], for which the association can be obtained by minimizing the following objective function Q*_j_*_2_, using an *L*_2_ penalty on top of Equation (3)

$$Q_{j2}^{}\left(\lambda_{\;j2}^{},\beta_{j1}^{},\cdots ,\beta_{\mathrm{jp}}^{}\right)=Q_{j0}^{}\left(\beta_{j1}^{},\cdots ,\beta_{\mathrm{jp}}^{}\right)+\lambda_{\;j2}^{}\sum_{\begin{array}{c} k=1 \end{array}}^{p}\beta_{\mathrm{jk}}^{2}$$

where *λ_j_*_2_ is the tuning (weight) parameter in the penalty term, *L*_2_, of Q*_j_*_2_.

Generally speaking, RRE is used to combat multi-collinearity owing to the shrinkage of inflation estimation variances arising from highly correlated gene expression data, while LASSO is used to exclude zero coefficients in large-scale regression-based GAN prediction through the adjustment of shrinkage parameters in the penalty term. More on penalized LS strategies have been discussed by [24,29].

### 2.3. An Alternative Parameter Estimation Method

In addition to these LS-based methods (LSE, RRE, and LASSO), an alternative, distribution-free estimation in regression models is the method of grouping estimators. Wald [30] proposed a special kind of grouping estimator called the Wald-type estimator (WTE) to tackle measuring noises (variations arising from measuring processes) in simple linear regression. Wald’s method divides the data into two groups according to predictor *X*: those above and below the observation median, respectively. The association parameters can then be estimated simply by computing the gradients of four means (those of the observed *X* and *Y* values, respectively, in the two divided groups). WTE has received little attention in the literature because it is inefficient as compared to LSE [31] and inconsistent with respect to measuring noises [32]. In addition, an assumption of independence between predictor variables is needed in multiple linear regression models, causing its poor performance in highly correlated data [17]. For more about WTE and methods of grouping, readers are referred to [31,33].

Recently, a generalized version of WTE called an adjusted Wald-type estimator (AWTE) has been developed to tackle Berkson-type uncertainties (i.e., noises in measurement but not errors caused by measuring process) and collinearity problems [17]. This non-parametric approach has several merits. First, for the multi-collinearity problem, AWTE is statistically consistent and asymptotically unbiased (overcoming the drawbacks of LSE, RRE and LASSO). Second, for the uncertainties in measurement error in conjunction with collinearities, whereas LSE may cause completely erroneous conclusions [34], AWTE can solve both problems simultaneously. It should be noted that, as Wu and Fang [17] pointed out, Berkson-type uncertainties are fundamentally different from the measuring noises discussed in [23,30] and are also different from the outliers treated in [25]: Namely, Berkson-type uncertainties can arise from biological noises while the other types are products of measuring processes. The application of AWTE to GAN construction will be formally described later.

### 2.4. Modeling Biological Noises and Correlated Expressions

Contributions from extrinsic and intrinsic noises in biological processes and correlated expressions may lead to biased regression modeling and incorrect predictions for a GAN [15,16]. To avoid such biases and to recover true associations, we consider the effects of both intrinsic and extrinsic noises in the framework of a linear regression system.

Let us begin with the consideration of intrinsic noises for not only the target gene *Y_j_* but also the predictor gene *X_k_* in Equation (2). As pointed out by Fujita and coworkers [23], the error term, *ε_yij_*, in the regression model can be seen as intrinsic noise in the expression of the target gene, *y_ij_*. However, the intrinsic noises of predictor genes, defined as *ε_xik_*’s below, are irrespective of measuring devices, although they also appear in measurements [35]. In other words, biological noises, which are Berkson-type uncertainties, can affect both the true and the observed expressions of target genes, while measuring noises such as those discussed in [23] affect only the observed expressions [36]. Therefore, if we would like to explicitly model intrinsic biological noise *ε_xik_* in predictor gene expression *x_ik_*, we can employ a Berkson-type uncertainty model [17] and rewrite Equation (2) as follows:

(4) $$y_{\mathrm{ij}}^{}=\sum_{\begin{array}{c} k=1 \end{array}}^{p}\beta_{\mathrm{jk}}^{}\left(x_{\mathrm{ik}}^{}+\varepsilon_{\mathrm{xik}}^{}\right)+\varepsilon_{\mathrm{yij}}^{}.$$

Next, to model extrinsic noises, it is suggested by [37] that a total noise should be identified, which can be the sum of intrinsic and extrinsic noises, and that these two types of noises should be presented separately to distinguish the contributions of their different origins. Thus, to account for noises of both intrinsic and extrinsic origins simultaneously in the regression system, we can rewrite Equation (4) as follows:

(5) $$y_{\mathrm{ij}}^{}=\sum_{k=1}^{p}\beta_{\mathrm{jk}}^{}\left(x_{\mathrm{ik}}^{}+\varepsilon_{\mathrm{xik}}^{}+v_{\mathrm{xik}}^{}\right)+v_{\mathrm{yij}}^{}+\varepsilon_{\mathrm{yij}}^{}$$

where the total noises in the expression of predictor gene *X_k_* and target gene *Y_j_* are *ε_xik_* + *v_xik_* and *ε_yij_* + *v_yij_*, respectively, in which *v_xik_* and *v_yij_* are extrinsic noises and *ε_xik_* and *ε_yij_* are intrinsic noises. Notice that the total noise for predictor gene *X_k_* may not influence target gene *Y_j_* if the association of these two genes is negligible, i.e., if the regression coefficient (*β_jk_*) is very close to zero. In contrast, the total noise for target gene *Y_j_* can cause its expression to fluctuate significantly whether or not the interactions between predictor and target genes are negligible. As a result, combining all noises into a single term in the modeling is problematic if the complexities of uncertainties are overly simplified.

Finally, to deal with the potential presence of collinearity, i.e., highly correlated gene expression data, we can assume that a predictor gene *X_l_* (*l* < *p*) is linearly dependent on another predictor gene *X_p_* (see [38] for a similar assumption about linear dependence between two genes); that is,

(6) $$\begin{array}{c} x_{i1}^{}=u_{i1}^{}+r_{1}^{}x_{\mathrm{ip}}^{},\\ x_{i2}^{}=u_{i2}^{}+r_{2}^{}x_{\mathrm{ip}}^{},\\ \;\;\;\;\;\vdots \\ x_{\mathrm{ip}-1}^{}=u_{\mathrm{ip}-1}^{}+r_{p-1}^{}x_{\begin{array}{c} ip \end{array}}^{}. \end{array}$$

Equation (6) intuitively divides gene expression *x_il_* (*l* < *p*) into two additive sources: the former source, *u_il_*, is a unique component for the predictor gene itself (i.e., independent of other genes) and the later source, *x_ip_*, is a common interaction component among *p* predictor genes and *r_l_* is their correlation parameter. Note that the framework of Equation (6) allows for ease of interpreting the structure of correlated observations and has been commonly used in the literature to address collinear configuration in regression analysis [17,39].

In summary, if highly correlated expression data and intracellular molecular noises are significant, WTE can be unstable, and conventional regression strategies (LSE, RRE, or LASSO) for deducing the values of association parameters *β*’s may be greatly biased. This is because specifying the exact means and variances of the total noise contributed from manifold origins is difficult, and as a result, the assumptions of the Gauss–Markov theorem do not hold. Furthermore, it is possible to over-adjust the penalty terms in Q*_j_*_1_ (for LASSO) or Q*_j_*_2_ (for RRE) for ill-conditioned problems due to the requirement of information on regression coefficients when estimating weight parameters.

### 2.5. A Robust Distribution-Free Regression Method for Modeling GAN Using AWTE

To consider the effects of biological noises on inferring a GAN, we can rewrite Equation (1) for the *i*-th independent experiment according to Equation (5)

(7) $$\begin{array}{c} y_{i1}^{}=\sum_{k=1}^{p}\beta_{1k}^{}\left(x_{\mathrm{ik}}+\varepsilon_{\mathrm{xik}}^{}+v_{\mathrm{xik}}^{}\right)+v_{\mathrm{yi}1}^{}+\varepsilon_{\mathrm{yi}1}^{}\\ y_{i2}^{}=\sum_{k=1}^{p}\beta_{2k}^{}\left(x_{\mathrm{ik}}+\varepsilon_{\mathrm{xik}}^{}+v_{\mathrm{xik}}^{}\right)+v_{\mathrm{yi}2}^{}+\varepsilon_{\mathrm{yi}2}^{}\\ \;\;\;\;\;\vdots \\ y_{\mathrm{iq}}^{}=\sum_{k=1}^{p}\beta_{\mathrm{qk}}^{}\left(x_{\mathrm{ik}}+\varepsilon_{\mathrm{xik}}^{}+v_{\mathrm{xik}}^{}\right)+v_{\mathrm{yiq}}^{}+\varepsilon_{\begin{array}{c} yiq \end{array}}^{} \end{array}$$

In addition, to deal with the influence of highly correlated data in regression models, the regressors can be constrained on Equation (6). To account for the complexity that may arise from stochastic noises of manifold origins, such as those described as non-Gaussian noises, we employed AWTE to obtain the association parameters *β*’s in Equation (7). The whole analytical procedure of the proposed distribution-free method, also referred to as AWTE, can be summarized by three primary steps.

*Step 1.* Determine the common interaction component (i.e., the second source of the additive combination, *x_ip_*) in Equation (6) among *p* predictor genes according to their observed expression levels; it can be made by

$$\underset{l<p}{\max}\rho^{2}\left(x_{p},x_{l}\right)>\underset{j\neq p}{\max}\underset{\begin{array}{c} k\neq j,p \end{array}}{\max}\rho^{2}\left(x_{j},x_{k}\right)$$

where *ρ* is the Pearson correlation coefficient and *x_k_* is the expression of gene *X_k_*.

*Step 2.* Estimate all the correlation parameters *r*_1_, *r*_2_, …, *r_p_*_−1_ in Equation (6), which can be achieved by

$${\hat{r}}_{l}^{}=\frac{\sum_{i=1}^{n}x_{\mathrm{il}}^{}\left(I\left[x_{\mathrm{ip}}^{}>M\left(x_{p}^{}\right)\right]-\frac{1}{2}\right)}{\sum_{\begin{array}{c} i=1 \end{array}}^{n}x_{\mathrm{ip}}^{}\left(I\left[x_{\mathrm{ip}}^{}>M\left(x_{p}^{}\right)\right]-\frac{1}{2}\right)}$$

where *l* = 1, …, *p*−1, *I* denotes the indicator function, i.e., *I*[A] = 1 if A is true, and 0 otherwise, **x***_k_* is an *n* × 1 vector with its *i*-th value equal to *x_ik_* for all *k* ≤ *p*, and *M*(**x***_k_*) is the median of all values in vector **x***_k_* (i.e., the median of *x*_1*k*_, *x*_2*k*_, …, *x_nk_*).

*Step 3.* Obtain the association parameter *β_jk_* in Equation (7) under the constraint of Equation (6), by using

(8) $${\hat{\beta }}_{\mathrm{jk}}^{}=\frac{\sum_{i=1}^{n}\left(y_{\mathrm{ij}}^{}-\tau_{\mathrm{jk}}^{}x_{\mathrm{ip}}^{}\right)\left(I\left[x_{\mathrm{ik}}^{}>{\hat{r}}_{k}^{}x_{\mathrm{ip}}^{}+M\left(x_{k}^{}-{\hat{r}}_{k}^{}x_{p}^{}\right)\right]-\frac{1}{2}\right)}{\sum_{\begin{array}{c} i=1 \end{array}}^{n}\left(x_{\mathrm{ik}}^{}-{\hat{r}}_{k}^{}x_{\mathrm{ip}}^{}\right)\left(I\left[x_{\mathrm{ik}}^{}>{\hat{r}}_{k}^{}x_{\mathrm{ip}}^{}+M\left(x_{k}^{}-{\hat{r}}_{k}^{}x_{p}^{}\right)\right]-\frac{1}{2}\right)}.$$

Equation (8) is the so-called AWTE, where $\hat{r}$_*p*_ is zero and *τ_jk_* is given by

$$\begin{array}{c} \tau_{\mathrm{jk}}^{}=\left\{\begin{array}{cc} 0 & k\neq p\\ \;\sum_{s=1}^{p-1}{\hat{r}}_{s}^{}{\hat{\beta }}_{\mathrm{js}}^{} & k=p \end{array}\right.. \end{array}$$

Note that, if we take all the values of $\hat{r}$_*k*_ to be zero, Equation (8) reduces to WTE.

A few remarks need to be made regarding the present approach. First, it has been pointed out by [17] that AWTE (Equation (8)) is a two-stage estimation method: estimating the whole set of regression coefficients except for the case of *k* = *p* first, then all the other regression coefficients by calculating *τ_jp_*’s. In this way, AWTE can be calculated directly without using iterative or optimization algorithms. Second, the computational cost of AWTE is *O*(*p*^2^) if *q* = 1 [17], and hence that of Equation (8) under Equation (6) is *O*(max[*p*^2^, *pq*]). In addition, as demonstrated in [17], by using this approach, we have theoretical guarantees for the robustness of the predicted GAN (see Appendix A for the theorem of robustness and its proof).

## 3. Results

In order to characterize the influences of expression noises on the performance of conventional LS-based regression methods (i.e., LSE, RRE and LASSO) and the present method (AWTE), we conducted a series of numerical simulations, in which levels of noises and sample sizes were varied to investigate the robustness of the networks constructed by these different methods.

### 3.1. Numerical Simulation Settings

Three common and standardized measures, power of detection (PD), false discovery rate (FDR) and inferential errors (INER), as suggested and defined in [6], were employed. Briefly summarizing, let B be the *p* × *q* parameter matrix of *β* in Equation (7). The three standardized measures are defined as follows: PD is the proportion of true associations (edges) in B detected; FDR is the proportion of predicted associations (edges) in B that are false detections; INER is the sum of all the deviations between estimated and true regression coefficients in B, where an edge between genes *Y_j_* and *X_k_* is regarded as detected if the absolute value of the estimate of association parameter *β_jk_* is greater than a cut-off value *τ*. We refer to [6] for detailed descriptions of these measures.

**Figure A2:**
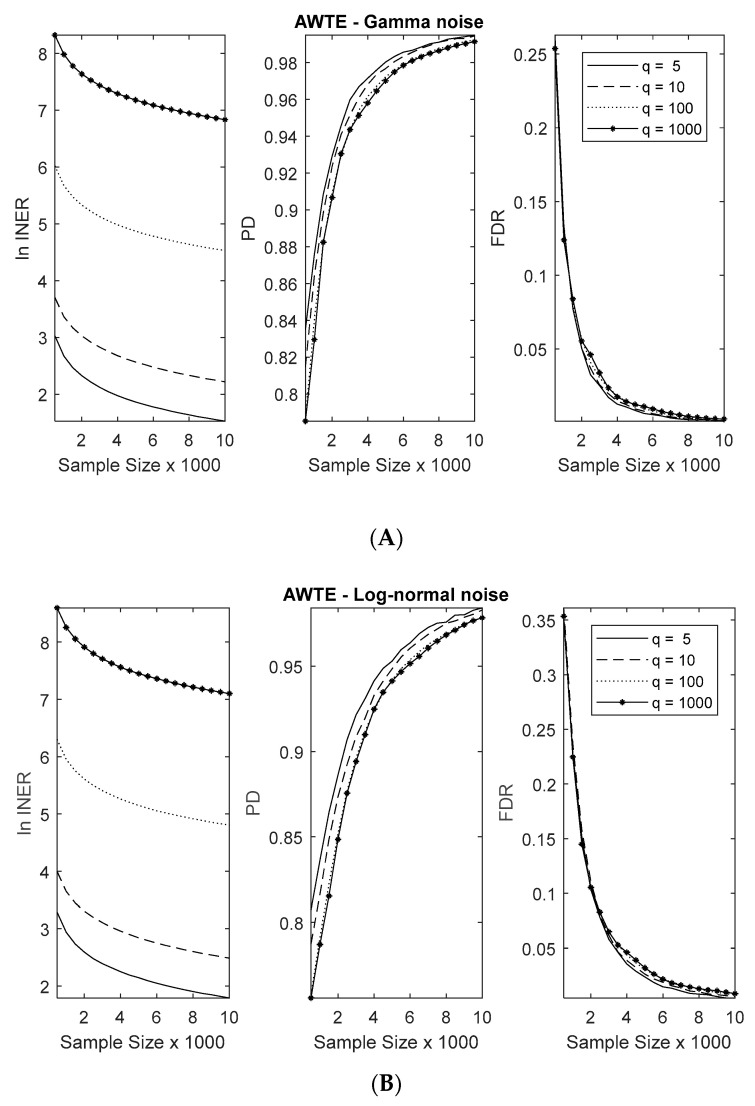
**The performance of AWTE as a function of target gene number *q*, using different types of non-Gaussian noise.** In each performance evaluation, 1000 repeated simulation runs were conducted using 10 predictor genes (*p* = 10) and a small noise level of *σ*^2^ = 1, and non-Gaussian noises generated by (**A**) gamma, and (**B**) log-normal, probability distribution function.

In these simulation experiments, we considered a system of ten predictor genes and five target genes (i.e., *p* = 10, *q* = 5) and a large set of observation samples, *n* ≥ 400. An association parameter *β_jk_* will be assigned a non-zero value chosen randomly from a uniformly distributed interval of [−1, −0.5] and [0.5, 1] with probability *π*, or set to zero otherwise with probability 1*−π*, where *π* can be regarded as the proportion of network edges that connect between X and Y genes. To avoid the situation in which all association parameters are zero, we set the regression coefficients of common interaction components, i.e., *β_jp_*’s for all *j* ≤ *q* in Equation (6), to be 0.9999. In addition, we assumed that in the same equation *u_il_* (*l* < *p*) and *x_ip_* in the additive combination of gene expression *x_il_* follow a chi-square distribution with a degree of freedom 2, and that random noises *ε_xik_*’s follow a chi-square distribution with a degree of freedom *σ*^2^ (*σ* being the level of noise) and random error terms *ε_yij_*’s follow a normal distribution with zero mean and 2*σ*^2^ variance. Note that the assumption of normal distribution for *ε_yij_*’s is commonly employed in other studies [6,23,40], and if we had used a non-Gaussian distribution for them, we would have obtained even larger errors for the conventional methods. Note also that although we used chi-square distributed intrinsic noises for predictor genes (*ε_xik_*’s) to synthesize gene expressions, the use of other types of non-Gaussian noises would not affect the conclusions of this study, because the robustness of our method has theoretical proof for noises of unknown probability density functions (see Appendix A) and this was buttressed in simulations using two other types of non-Gaussian noises (Appendix B Figure A2).

For simplicity, the common interaction component among *p* predictor genes was assumed to be known because it can be identified from Step 1 of the AWTE procedure. To mimic the influences of the many sorts of extrinsic noises, we furthermore assumed that the extrinsic noises *v_yij_* and *v_xik_* in Equation (7) were replaced by non-linear functions *f_Y_*(*v_yij_*) and *f_X_*(*v_xik_*), respectively, where we let *f_Y_*(*v*) = *v*^2^ + 2sin(*v*) and *f_X_*(*v*) = cos(*v*), with *v_yij_*’s and *v_xik_*’s sharing the same probability density function of *ε_xik_*. Note that in our method, the distributions of observed expressions and noises, hence the non-linear functions *f_Y_* and *f_X_*, need not be known, but they need to be specified in a certain form in order to generate the synthetic data needed for the simulations.

### 3.2. Method Comparisons in Numerical Simulations

**Figure 1:**
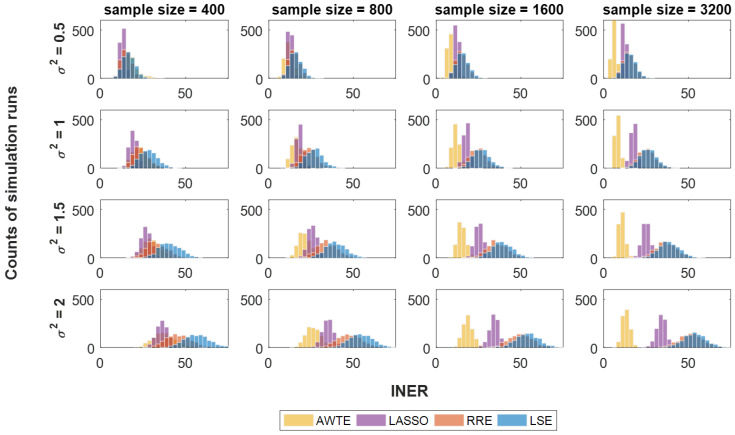
**Results of INER at various levels of noises and sample sizes for four different regression-based methods.** In each histogram, 1000 repeated simulation runs were conducted for each method, with the INER performance (*X*-axis) color-coded yellow for AWTE, purple for LASSO, orange for RRE, and blue for LSE. Data with increasing levels of noise are indicated by increasing values of *σ*^2^ shown to the left of simulation run counts (*Y*-axis).

**Figure 2:**
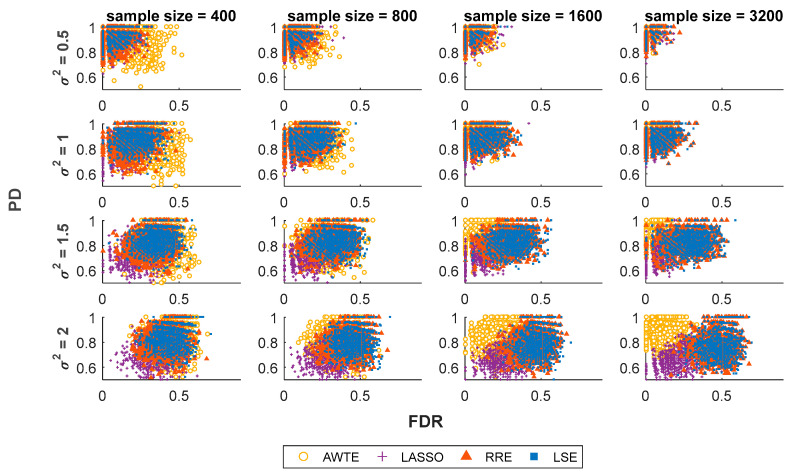
**Results of PD and FDR at various levels of noise and sample sizes for four different regression-based methods.** In each scatter plot, 1000 repeated simulation runs were conducted for each method, with the results of PD (*Y*-axis) and FDR (*X*-axis) indicated by yellow circles for AWTE, purple pluses for LASSO, orange triangles for RRE, and blue squares for LSE. Data with increasing levels of noise are indicated by increasing values of *σ*^2^ shown to the left of the *Y*-axis, where an optimal cut-off point was selected via ROC curve analysis to predict an association.

**Figure 3:**
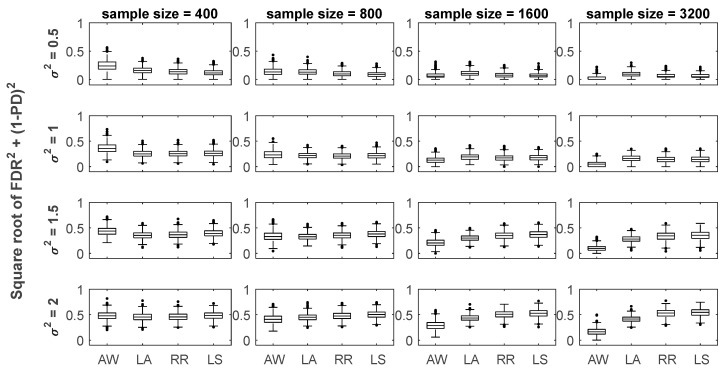
**Results of FDR/PD-combined performance at various levels of noise and sample sizes for four different regression-based methods**. In each box plot, the median values of the square root of FDR^2^ + (1 − PD)^2^ were compared for the combined performance. Within each plot, the results for AWTE (AW), LASSO (LA), RRE (RR), and LSE (LS) are shown from left to right. Data with increasing levels of noise are indicated by increasing values of *σ*^2^ shown to the left of the *Y*-axis, where an optimal cut-off point was selected via ROC curve analysis to predict an association.

Based on the settings and the frameworks described in Section 3.1 and Equations (6) and (7) in Section 2, we numerically generated gene expression data and used them for a series of 1000 repeated simulation runs with prescribed parameter values representing different levels of noises and sample sizes. From these simulations, measures of PD (power of detection), FDR (false discovery rate) and INER (inferential error) were computed in the receiver operating characteristic (ROC) curve analysis in which an optimal cutoff point for best performance was determined. We evaluated the resulting predicted GANs using these standard statistical measures in Figure 1, Figure 2 and Figure 3 where *r_l_* = 1.5 (i.e., Pearson correlation coefficient between genes *X_l_* and *X_p_* was set to be greater than 0.8, which indicates a condition of high correlation; see Equation (6)) and *π* = 0.4 (the ratio of network connectivities that are truly associated; see Section 3.1) were fixed to examine the effects of different levels of noises (indicated by *σ*^2^) and sample sizes. These allowed us to evaluate the performance and robustness of different methods under conditions of high collinearities.

As expected and shown by Figure 1 and Figure 2, higher levels of noise led to larger values of INER and FDR for all the four methods investigated. However, compared to three conventional regression methods, LASSO [27], RRE [28] and LSE [26], our method (AWTE) was significantly less sensitive to increasing levels of noises for both INER (Figure 1) and FDR (Figure 2), especially as the sample size increased. Since PD, the proportion of correctly inferred network edges for all non-zero association parameters (*β*), can be high even when FDR is also high, the two need to be evaluated together. A combined measure, the square root of FDR^2^ + (1 − PD)^2^, was therefore used, and the results are shown in Figure 3. As can be seen, when the sample size was small, AWTE performed somewhat worse than the three conventional methods when the noise level was low, but as the sample size increased, AWTE gradually gained an advantage and then significantly outperformed the conventional methods when the noise level was high. Notably, the GAN was inferred within 20 s on a general-purpose PC equipped with Intel CPU i7-4790 and 8 GB of RAM for a total of 1000 simulation runs using AWTE; similar timings were obtained with RRE and LSE, but LASSO took over 300 min to complete the same task.

**Figure 4:**
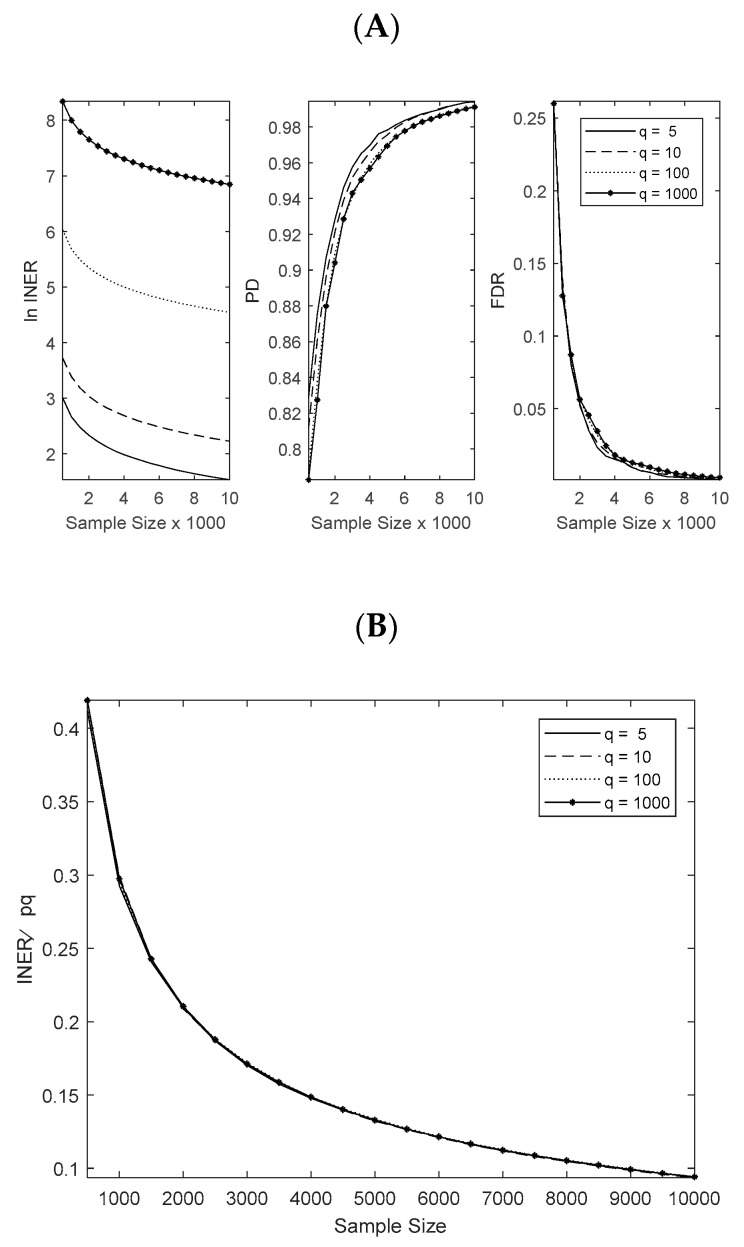
**Performance of AWTE at various target gene numbers (*q*) as a function of sample size based on ROC curve analysis.** In each performance evaluation, 1000 repeated simulation runs were conducted (using *p* = 10 and a small level of noise *σ*^2^ = 1), where an optimal cut-off point was selected via ROC curve analysis to predict an association. (**A**) Results for the natural logarithm of INER (left), PD (middle) and FDR (right). (**B**) Results for INER/*pq*, the average estimation deviation of the association parameters, *β*’s.

**Figure A3:**
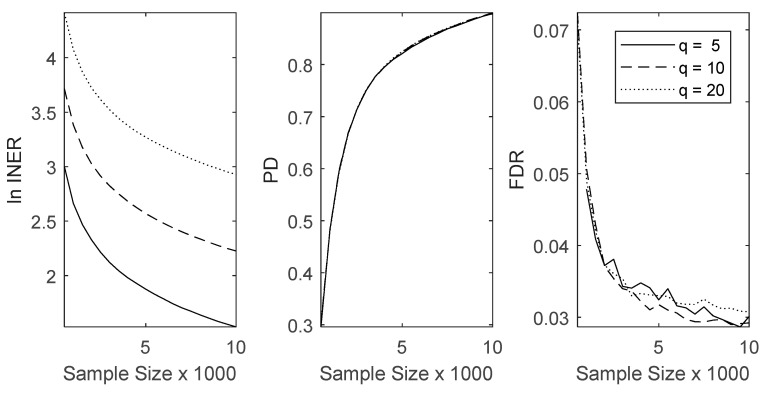
**The performance of AWTE as a function of sample size and varying number (*q*) of target genes, with associations inferred by statistical test of significance.** In this evaluation, 1000 repeated simulation runs were conducted using 10 predictor genes (*p* = 10) and a small level of noise *σ*^2^ = 1, and a standard *p*-value cutoff (0.05) was used to infer an association. Because computing all the *p*-values is time consuming, the simulations were conducted only for *q* ≤ 20.

To gain further insight into the theoretical behaviors of the proposed approach, simulations using a broader range of sample sizes and different scales of simultaneous expression equations (*q* = 5, 10, 100, 1000) were conducted and analyzed. A typical result is shown in Figure 4 (also see Appendix B Figure A2), where a low level of noise (*σ*^2^ = 1) was applied, and the number of predictor genes, X, was set to be 10 (*p* = 10). As can be seen from Figure 4A, estimation deviations, as indicated by INER, decreased as the sample size increased. Furthermore, as shown in Figure 4B, although INER increased with an increased number (*q*) of target genes (Figure 4A), the average estimation deviation of *β*, i.e., INER/*pq*, remained nearly constant at any given sample size for all the numbers of target genes tested and decreased as the sample size increased. These results confirm the Theorem A1 (see Appendix A) and demonstrate the robustness of AWTE. These results also indicate that a desirable outcome (e.g., with PD > 0.95 and FDR < 0.05) can be expected using AWTE when a sufficiently large number of observations (e.g., sample size *n* > 3000) are available. Importantly, all these statistical behaviors remained true when a standard *p*-value cutoff (0.05) was used to predict an association (Appendix B Figure A3), although the results for PD were somewhat worse than those shown in Figure 4 where a threshold value for the association parameters was selected to produce the best ROC performance. This is an important point to make because it demonstrates the potential of AWTE to reliably predict gene associations from gene expression data in the absence of literature knowledge on those associations.

### 3.3. Method Comparisons Using an Actual Lymphoma Dataset

To evaluate the potential of the proposed method for practical applications, we tested it on a known network of TF (transcription factor) genes and the genes they regulate in the germinal-center regulatory program of B cells. As reviewed in [41], dysregulation of this network is a cause of many types of lymphomas. In this case, a study using actual gene expression data of lymphoma, we would like to find out to what extent the experimentally documented associations of this GAN of germinal center B-cell genes can be predicted by AWTE, in comparison to conventional methods.

We retrieved the gene expression data of diffuse large B-cell lymphoma (DLBCL) contributed by [42] from Gene Expression Omnibus (GEO, [43]). This dataset (GEO: GSE60) contained data for five (*BCL6*, *BACH2*, *SPIB*, *IRF8*, and *OCT2*) of the seven TF genes (modeled as predictor genes) and all the ten target genes (*p21*, *MYC*, *P53*, *BCL2*, *NFKB1*, *IRF4*, *Blimp1*, *AID*, *p27*, and *ATR*) of the germinal center regulation network described in Figure 1 of [41]. The data of the total sample size (N = 133) from GSE60 for both normal cells (N = 31) and tumor cells (N = 102) containing both GCB (germinal center B-cell) and ABC (activated B-cell) subtypes were retrieved and analyzed.

**Figure 5:**
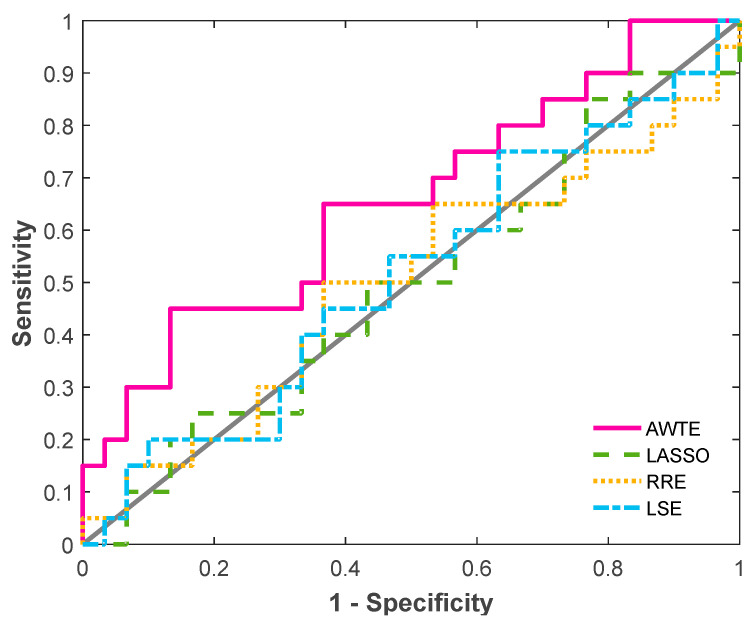
**ROC curve analysis for four different regression-based methods for the case study of germinal center B cell gene associations.** The ROC curves made by each of the four regression-based methods were plotted, yielding an AUC value of 0.66 for AWTE, 0.50 for LASSO, 0.50 for RRE, and 0.51 for LSE.

The AWTE-produced association parameters for the network (a 5 × 10 matrix) of this dataset are presented in Table A1 (Appendix B), and the ROC performances for AWTE, LASSO, RRE, and LSE are shown in Figure 5. The ROC performances were determined by treating the experimentally observed associations (dash-boxed in Table A1) as real and all the rest as nonexistent—ignoring the fact that absence of observation does not necessarily equate to the absence of association. As can be seen in Figure 5, AWTE, having the largest area under the ROC curve (AUC), significantly outperformed the three conventional methods, which did not perform better than random guesses (the diagonal line in the ROC plot). Interestingly, of the several associations predicted using AWTE with statistical significance (*p*-value < 0.05; bold-typed in Table A2) but have not yet been verified by human data, three (asterisked in Table A1) can find support from mouse studies: SPIB-AID [44], BACH2-AID [45], and IRF8-IRF4 [46]. In summary, a gene trio motif of gene regulation could be clearly identified in Table A1 based on the statistical significance of the gene associations deduced: namely, SPIB, BACH2, and OCT2 are regulators of oncogenes IRF4 and AID.

The results obtained with models of three conventional regression methods were presented in Table A3, Table A4 and Table A5. Generally speaking, the patterns of their GANs were quite similar to ours, but notable differences existed. For example, both IRF4 and AID were down-regulated by BACH2 in the AWTE-inferred GAN, but only AID was in the LS-inferred GAN. In addition, whereas the down-regulation of IRF4 by BCL6 was evident in both GANs, the up-regulation of AID by BCL6 was significant only in the LS-inferred GAN. In combination, these results appear to reflect the experimental observation of the tumor suppressor role of BACH2 [47] and the dual regulation role of BCL6 [41].

## 4. Discussion

**Figure A4:**
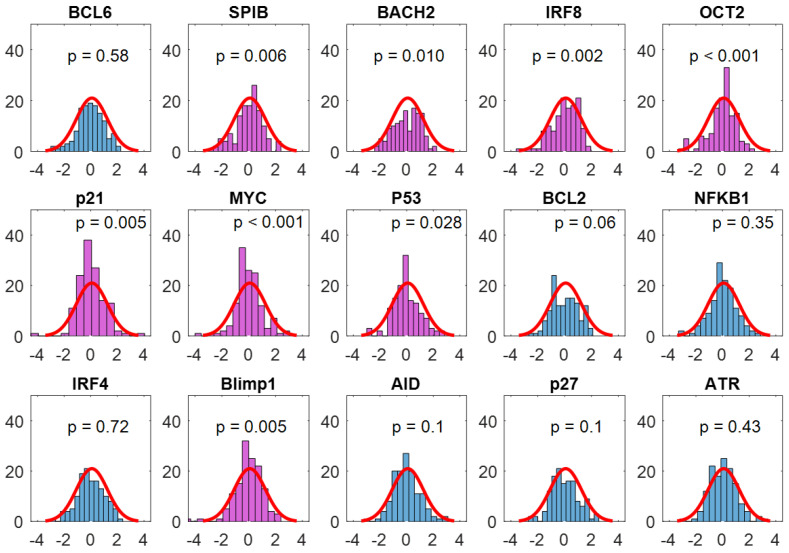
**Results of Anderson–Darling normality test for the expressions of each of the 15 B-cell genes (Table A1) examined among the 133 samples of the GSE60 lymphoma dataset.** Histograms in pink are the genes exhibiting a statistically significant deviation from normal distribution (*p*-value < 0.05), and in blue are those not. Standard normal distribution is drawn by red curve in each histogram.

The LS strategy, which is mathematically equivalent to the maximum likelihood estimation, is known to perform well for systems with Gaussian noises, i.e., noises that are characterized by normal distributions [26]. However, when noises are non-Gaussian, LS-based methods can be unsatisfactory. For example, as can be seen in Figure 1, when the sample size is as large as 3200, conventional methods can be unstable under conditions of non-Gaussian noises, with LSE and RRE having a wide range of INER even when the level of noise is not high (e.g., *σ*^2^ = 1). In addition, as can be seen from Figure 3 at sample size = 3200, LASSO performed better (lower median in the box plot) than LSE and RRE in the case of *σ*^2^ > 1 but worse in the case of *σ*^2^ ≤ 1, which suggests that in this example LASSO failed the test of robustness. Taken together, we can conclude that, as did others [15,48], a predicted gene network might be non-functional (e.g., with high INER and high FDR values) or even incorrect if the effects of intrinsic or extrinsic noises are ignored or overly simplified to reduce analysis complexities. Indeed, the expressions of eight of the fifteen genes analyzed in Table A1 for lymphoma did not pass the normality test (Appendix B Figure A4), which could be a reason for the poor ROC results of the LS-based methods for predicting the B-cell GAN (Figure 5).

**Figure A5:**
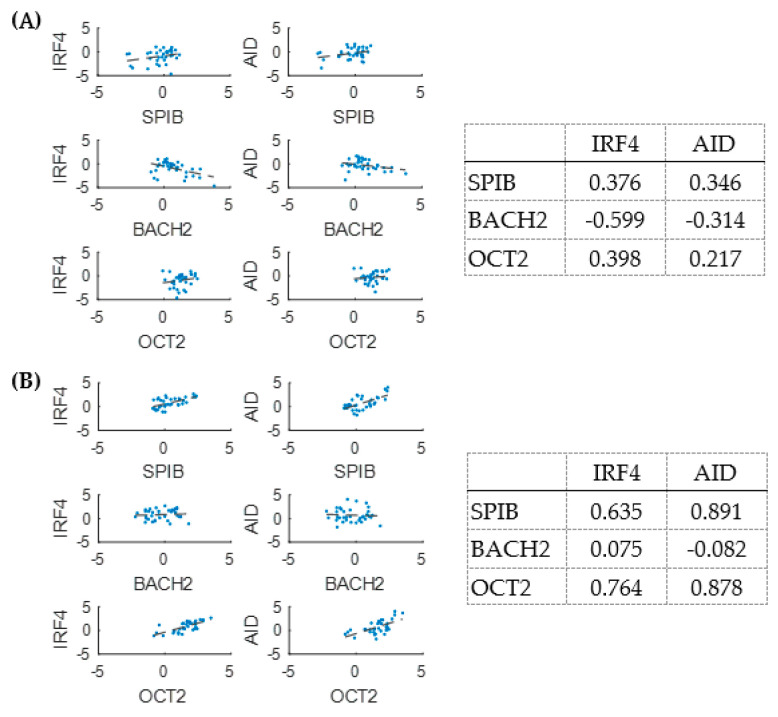
**Scatter plots of gene expression levels and their linear trends for the proposed gene trio motif in GSE60 DLBCL (A) GCB subtype (N = 35) and (B) ABC subtype (N = 35).** In each subtype, scatter plot for each pair in the gene trio motif was drawn. Linear trends for regulating roles in the gene trio motif were also analyzed using simple linear regressions (3-by-2 table, right hand side), where negative (positive) slope indicates down- (up-) regulations and a flat slope (close to zero) indicates limited regulations.

To assess the potential use of the gene trio regulation motif for practical applications, we conducted a subtype analysis in DLBCL GCB and ABC using data from GSE60. As may be seen in Figure A5A, the regulations of the motif, as suggested in GCB patients’ gene expression data, are consistent with the overall trend of our model (Table A1). However, in Figure A5B), the down-regulating function of BACH2 is nearly non-existent, while the up-regulating function of SPIB and OCT2 to the two oncogenes in the ABC subtype is stronger compared to the GCB subtype. Although we do not know specific mechanisms of how these TF genes can help differentiate the subtypes, these observations may suggest that over-expression of SPIB and OCT2, as well as malfunction of BACH2, could be probable causes leading to higher IRF4/AID expressions and resulting in different clinical outcomes for patients with different subtypes.

In the present work, we did not consider measuring noises because their modeling may require additional experimental data and/or analysis procedures, as well as a distribution-dependent approach (e.g., [23]). It is a problem not within the scope of the present study but will be a subject of our future research.

There are a few other limitations of our method in its current form. Firstly, although a wider range of design specifications can be used to construct GANs because AWTE can model uncertain noises with fewer constraints, our method may not perform as well as conventional LS-based methods if the number of observations is not sufficiently large, as Figure 1, Figure 2 and Figure 3 indicate. However, array-based experiments and other high-throughput technologies to produce very large expression datasets have become increasingly available in recent years, as in studies using TCPA (The Cancer Proteome Atlas, [49]; sample size > 3000), TCGA (The Cancer Genome Atlas, [50]; sample size > 800) or UK biobank ([51]; sample size of 500,000 around), this limitation of sample size may soon become a non-issue in many applications.

Secondly, if in the model the number of predictor genes, *p*, is larger than that of experiments, *n*, overfitting may occur, which is a major statistical limitation of linear regression analysis [24]. To circumvent this problem, automatic variable selection techniques (e.g., stepwise, forward, or backward selections) can be potentially helpful to screen for favorite predictor genes so as to consider only a smaller number of them (i.e., *n* > *p*) in applying the proposed approach. Or, as demonstrated by the case study of lymphoma in the present work, knowledge and information from the literature, despite being far from complete and often not straightforward, can be harnessed for the new method to make insightful discoveries on gene regulations.

Thirdly, we did not consider time series data mainly because regression modeling for time series observations often requires distribution-dependent procedures (see, e.g., [52]) or a distribution-free procedure as in the work of [53], for which, however, theoretical justifications are still lacking to prove that a generalized LS-based approach can address well the manifold uncertainties associated with the predictors of interest. Further studies are required to fully address this statistical issue.

**Figure A6:**
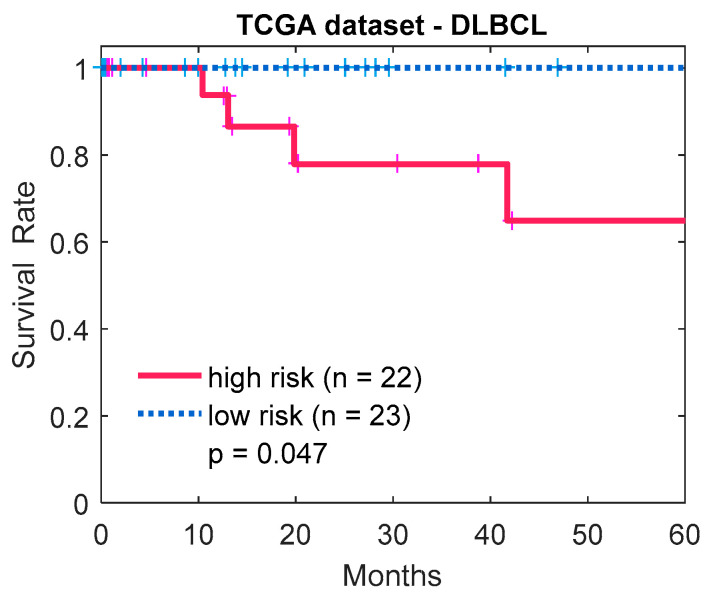
**Survival analysis using gene expressions of gene trio, SPIB, BACH2 and OCT2, as a prognostic signature.** DLBCL dataset was retrieved from TCGA [50]. Risk groups were stratified at median of risk scores calculated via Cox modeling.

Fourthly, our model was derived from data from an older array platform, which may cause biases in the analysis and hence reduce the accuracy of the results. The predictive value of the gene trio motif has also not been fully investigated, although in a preliminary analysis we found that the trio can be a prognostic signature to distinguish survival risks of lymphoma patients (Figure A6). Further validation with newer data of the model and the gene trio motif in cancer gene regulation is ongoing.

Finally, our method in the present work was applied to only a handful of variables (genes). In principle, one could consider all TFs as predictor genes to regulate all other genes and build a whole-genome TF-centered GAN. However, it remains to be investigated if the existing data are sufficient to overcome overfitting for such an undertaking. A strategy such as principal component analysis to shrink the dimension of these TFs while keeping all the data in the analysis may be necessary.

## Data Availability

All of the data analyzed in the present work are available from public domain as stated in the main text. MATLAB codes supporting the results in the paper are available upon reasonable request from the corresponding author (W.-Q.F.).

## Appendix A

Theoretical property and its proof of robustness concerning statistical consistency and unbiasedness for AWTE are summarized as follows.

**Theorem** **A1.**
*For the distribution-free regression approach with respect to predicting the GAN in Equation (7), suppose that it is constrained on Equation (6), and that, in Equation (6), we can specify the common interaction component among p predictor genes based on their observed expression levels, if the whole design specifications (D1)–(D5), described below, are satisfied, then estimations of the association parameters in Equation (8), i.e., the regression coefficients, are consistent and (approximately) unbiased statistically.*

(D1) The intrinsic noise of *y_j_* (i.e., *ε_y_*_1*j*_, *ε_y_*_2*j*_, …, *ε_ynj_*), the intrinsic noise of *x_k_* (i.e., *ε_x1k_*, *ε_x2k_*, …, *ε_xnk_*), the extrinsic noise of *y_j_* (i.e., *v_y1j_*, *v_y2j_*, …, *v_ynj_*), and the extrinsic noise of *x_k_* (i.e., *v_x1k_*, *v_x2k_*, …, *v_xnk_*), in regression model (7), for all positive integers *j* and *k*, are all independently and identically sampled random variables. Furthermore, their fourth moments are finite; that is,
  $$\underset{j\leq q}{\max}\left\{\int \varepsilon_{}^{4}g_{\mathrm{yj}}(\varepsilon )d\varepsilon \right\}+\underset{k\leq p}{\max}\left\{\int \varepsilon_{}^{4}g_{\mathrm{xk}}(\varepsilon )d\varepsilon \right\}<\infty \mathrm{and}\;\underset{j\leq q}{\max}\left\{\int v_{}^{4}h_{\mathrm{yj}}(v)\mathrm{dv}\right\}+\underset{k\leq p}{\max}\left\{\int v_{}^{4}h_{\mathrm{xk}}(v)\mathrm{dv}\right\}<\infty$$
  where *g_yj_* and *g_xk_* are unknown probability density functions of the intrinsic noises of *y_j_* and *x_k_*, respectively, and *h_yj_* and *h_xk_* are unknown probability density functions of the extrinsic noises of *y_j_* and *x_k_*, respectively.

(D2) The expressions of unique component for predictor gene *X_l_* (i.e., *u*_1*l*_, *u*_2*l*_, …, *u_nl_* for *l* < *p*) and those of common interaction component (i.e., *x*_1*p*_, *x*_2*p*_, …, *x_np_*) in Equation (6) are all independently and identically sampled random variables. Furthermore, their fourth moments are finite; that is,
  $$\underset{l<p}{\max}\left\{\int u_{}^{4}f_{\mathrm{ul}}(u)\mathrm{du}\right\}+\int x_{}^{4}f_{\mathrm{xp}}(x)\mathrm{dx}<\infty$$
  where *f_ul_* and *f_xp_* are unknown probability density functions of *u_il_* and *x_ip_*, respectively.

(D3) The observed gene expressions *x*_1*p*_, *x*_2*p*_, …, *x_np_* and the expressions of unique component *u*_1*l*_, *u*_2*l*_, …, *u_nl_* for predictor gene *X_l_* in Equation (6), for all positive integers *l* < *p*, are independent of intrinsic and extrinsic noises.
(D4) The expressions of unique component *u*_1*t*_, *u*_2*t*_, …, *u_nt_* for predictor gene *X_t_* are independent of those of all the other unique component *u*_1*l*_, *u*_2*l*_, …, *u_nl_* and common interaction component *x*_1*p*_, *x*_2*p*_, …, *x_np_* in Equation (6), for all *t* not equal to *l*.
(D5)$\sum_{\begin{array}{c} i=1 \end{array}}^{n}\left(x_{\mathrm{ik}}^{}-cx_{\mathrm{ip}}^{}\right)\left(I\left[x_{\mathrm{ik}}^{}>cx_{\mathrm{ip}}^{}+M\left(x_{k}^{}-cx_{p}^{}\right)\right]-\frac{1}{2}\right)>0$, for all real number *c* except for the case of *c* = 1 and *k* = *p*.

**Proof of** **Theorem A1.** Below, for the convenience of describing the mathematical proofs, we shall define a few nomenclatures. Let |.| denote the absolute value of a real number (or a random variable) and *p*(A) denote the probability of an event (or a set) A.

First, we will prove the statistical consistency of Equation (8); that is, for an arbitrary positive number *ε*, we need to show

(A1) $$\underset{n\to \infty }{\lim}p\left(\underset{j,k}{\max}\left|{\hat{\beta }}_{\mathrm{jk}}^{}-\beta_{\mathrm{jk}}^{}\right|>\varepsilon \right)=0.$$

Let *R_Ut_*, *R_ΕXt_*, *R_VXt_*, *R_ΕYj_* and *R_VYj_* be defined as follows:

$$R_{\mathrm{Ut}}^{}=\frac{\sum_{i=1}^{n}u_{\mathrm{it}}^{}\left(I\left[x_{\mathrm{ip}}^{}-M\left(x_{p}^{}\right)>0\right]-\frac{1}{2}\right)}{\sum_{i=1}^{n}x_{\mathrm{ip}}^{}\left(I\left[x_{\mathrm{ip}}^{}-M\left(x_{p}^{}\right)>0\right]-\frac{1}{2}\right)},\;R_{\mathrm{EXt}}^{}=\frac{\sum_{i=1}^{n}\varepsilon_{\mathrm{xit}}^{}\left(I\left[x_{\mathrm{ip}}^{}-M\left(x_{p}^{}\right)>0\right]-\frac{1}{2}\right)}{\sum_{i=1}^{n}x_{\mathrm{ip}}^{}\left(I\left[x_{\mathrm{ip}}^{}-M\left(x_{p}^{}\right)>0\right]-\frac{1}{2}\right)},\;R_{\mathrm{VXt}}^{}=\frac{\sum_{i=1}^{n}v_{\mathrm{xit}}^{}\left(I\left[x_{\mathrm{ip}}^{}-M\left(x_{p}^{}\right)>0\right]-\frac{1}{2}\right)}{\sum_{i=1}^{n}x_{\mathrm{ip}}^{}\left(I\left[x_{\mathrm{ip}}^{}-M\left(x_{p}^{}\right)>0\right]-\frac{1}{2}\right)},\;R_{\mathrm{EYj}}^{}=\frac{\sum_{i=1}^{n}\varepsilon_{\mathrm{yij}}^{}\left(I\left[x_{\mathrm{ip}}^{}-M\left(x_{p}^{}\right)>0\right]-\frac{1}{2}\right)}{\sum_{i=1}^{n}x_{\mathrm{ip}}^{}\left(I\left[x_{\mathrm{ip}}^{}-M\left(x_{p}^{}\right)>0\right]-\frac{1}{2}\right)},\;R_{\mathrm{VYj}}^{}=\frac{\sum_{i=1}^{n}v_{\mathrm{yij}}^{}\left(I\left[x_{\mathrm{ip}}^{}-M\left(x_{p}^{}\right)>0\right]-\frac{1}{2}\right)}{\sum_{i=1}^{n}x_{\mathrm{ip}}^{}\left(I\left[x_{\mathrm{ip}}^{}-M\left(x_{p}^{}\right)>0\right]-\frac{1}{2}\right)}.$$

Observe that

$$\begin{array}{c} \underset{k}{\max}\left|{\hat{\beta }}_{\mathrm{jk}}^{}-\beta_{\mathrm{jk}}^{}\right|\leq \sum_{l<p}^{}\left|{\hat{\beta }}_{\mathrm{jl}}^{}-\beta_{\mathrm{jl}}^{}\right|+\left|\tau_{\mathrm{jp}}^{}-\sum_{s<p}^{}r_{s}^{}\beta_{\mathrm{js}}^{}\right|+\left|\sum_{t<p}^{}\left(R_{\mathrm{Ut}}^{}+R_{\mathrm{EXt}}^{}+R_{\mathrm{VXt}}^{}\right)\beta_{\mathrm{jt}}^{}\right|\\ +\left|R_{\mathrm{VYj}}^{}+R_{\mathrm{EYj}}^{}\right|\;\\ \;\;\;\;\leq \sum_{l<p}^{}\left|{\hat{\beta }}_{\mathrm{jl}}^{}-\beta_{\mathrm{jl}}^{}\right|\left(1+\left|{\hat{r}}_{l}^{}\right|\right)+\sum_{s<p}^{}\left|\beta_{\mathrm{js}}^{}\right|\left|{\hat{r}}_{s}^{}-r_{s}^{}\right|+\sum_{t<p}^{}\left|\beta_{\mathrm{jt}}^{}\right|\left|R_{\mathrm{Ut}}^{}+R_{\mathrm{EXt}}^{}+R_{\mathrm{VXt}}^{}\right|\\ +\left|R_{\mathrm{VYj}}^{}\right|\;+\left|R_{\mathrm{EYj}}^{}\right| \end{array}$$

This implies that

(A2) $$\begin{array}{c} p\left(\underset{k}{\max}\left|{\hat{\beta }}_{\mathrm{jk}}^{}-\beta_{\mathrm{jk}}^{}\right|>\frac{\varepsilon }{q}\right)\leq \sum_{l<p}^{}p\left(\left|{\hat{\beta }}_{\mathrm{jl}}^{}-\beta_{\mathrm{jl}}^{}\right|>\frac{\varepsilon }{\left(1+\underset{s}{\max}r_{s}^{*}\right)\left(3p-1\right)q}\right)\\ \;\;\;\;\;\;\;\;\;\;\;\;\;\;\;\;\;\;+\sum_{s<p}^{}p\left(\left|{\hat{r}}_{s}^{}-r_{s}^{}\right|>\frac{\varepsilon }{\left(\underset{l}{\max}\left|\beta_{\mathrm{jl}}^{}\right|\right)\left(3p-1\right)q}\right)\\ \;\;\;\;\;\;\;\;\;\;\;\;\;\;\;\;\;\;\;\;\;\;\;\;\;\;+\sum_{t<p}^{}p\left(\left|R_{\mathrm{Ut}}^{}+R_{\mathrm{EXt}}^{}+R_{\mathrm{VXt}}^{}\right|>\frac{\varepsilon }{\left(\underset{l}{\max}\left|\beta_{\mathrm{jl}}^{}\right|\right)\left(3p-1\right)q}\right)\\ \;\;\;\;\;\;\;\;\;\;\;\;\;\;\;\;\;\;\;\;\;\;\;\;+p\left(\left|R_{\mathrm{EYj}}^{}\right|>\frac{\varepsilon }{\left(3p-1\right)q}\right)+p\left(\left|R_{\mathrm{VYj}}^{}\right|>\frac{\varepsilon }{\left(3p-1\right)q}\right) \end{array}$$

where $r_{s}^{*}$ is the maximal value of |${\hat{r}}_{s}^{}$| for all sample size *n*. According to the assumption of independent and identical sampling made in design specifications D1–D4, it immediately follows from Lemma-(i) and the similar arguments of (A.2) and (A.3) in [17] that, for arbitrary positive numbers *λ_m_*, *m* = 1, 2, …, 8,

(A3) $$\underset{n\to \infty }{\lim}p\left(\left|{\hat{\beta }}_{\mathrm{jl}}^{}-\beta_{\mathrm{jl}}^{}\right|>\lambda_{\;1}^{}\right)=\underset{n\to \infty }{\lim}p\left(\left|{\hat{r}}_{s}^{}-r_{s}^{}\right|>\lambda_{\;2}^{}\right)=0$$

and that

(A4) $$\underset{n\to \infty }{\lim}p\left(\left|R_{\mathrm{EYj}}^{}\right|>\lambda_{\;3}^{}\right)=\underset{n\to \infty }{\lim}p\left(\left|R_{\mathrm{VYj}}^{}\right|>\lambda_{\;4}^{}\right)=0$$

and that

$$\underset{n\to \infty }{\lim}p\left(\left|R_{\mathrm{Ut}}^{}\right|>\lambda_{\;5}^{}\right)=\underset{n\to \infty }{\lim}p\left(\left|R_{\mathrm{EXt}}^{}\right|>\lambda_{\;6}^{}\right)=\underset{n\to \infty }{\lim}p\left(\left|R_{\mathrm{VXt}}^{}\right|>\lambda_{\;7}^{}\right)=0$$

which yields

(A5) $$\underset{n\to \infty }{\lim}p\left(\left|R_{\mathrm{Ut}}^{}+R_{\mathrm{EXt}}^{}+R_{\mathrm{VXt}}^{}\right|>\lambda_{\;8}^{}\right)=0.$$

Observe that

$$p\left(\underset{j,k}{\max}\left|{\hat{\beta }}_{\mathrm{jk}}^{}-\beta_{\mathrm{jk}}^{}\right|>\varepsilon \right)\leq \sum_{j\leq q}^{}p\left(\underset{k}{\max}\left|{\hat{\beta }}_{\mathrm{jk}}^{}-\beta_{\mathrm{jk}}^{}\right|>\frac{\varepsilon }{q}\right).$$

Hence, based on (A2)–(A5), and let *ε*^*^ = *ε/*(3*pq* − *q*), we have

$$\begin{array}{c} \underset{n\to \infty }{\lim}p\left(\underset{j,k}{\max}\left|{\hat{\beta }}_{\mathrm{jk}}^{}-\beta_{\mathrm{jk}}^{}\right|>\varepsilon \right)\leq \sum_{j\leq q}^{}\underset{n\to \infty }{\lim}p\left(\underset{k}{\max}\left|{\hat{\beta }}_{\mathrm{jk}}^{}-\beta_{\mathrm{jk}}^{}\right|>\frac{\varepsilon }{q}\right)\\ \;\;\;\;\;\;\;\;\;\;\;\;\;\;\;\;\;\leq \sum_{j\leq q}^{}\sum_{l<p}^{}\underset{n\to \infty }{\lim}p\left(\left|{\hat{\beta }}_{\mathrm{jl}}^{}-\beta_{\mathrm{jl}}^{}\right|>\frac{\varepsilon_{}^{*}}{\left(1+\underset{s}{\max}r_{s}^{*}\right)}\right)\\ \;\;\;\;\;\;\;\;\;\;\;\;\;\;\;\;\;+q\sum_{s<p}^{}\underset{n\to \infty }{\lim}p\left(\left|{\hat{r}}_{s}^{}-r_{s}^{}\right|>\frac{\varepsilon_{}^{*}}{\underset{j,\;l}{\max}\left|\beta_{\mathrm{jl}}^{}\right|}\right)\\ \;\;\;\;\;\;\;\;\;\;\;\;\;\;\;\;\;\;\;\;\;+q\sum_{t<p}^{}\underset{n\to \infty }{\lim}p\left(\left|R_{\mathrm{Ut}}^{}+R_{\mathrm{EXt}}^{}+R_{\mathrm{VXt}}^{}\right|>\frac{\varepsilon_{}^{*}}{\underset{j,\;l}{\max}\left|\beta_{\mathrm{jl}}^{}\right|}\right)\\ \;\;\;\;\;\;\;\;\;\;\;\;\;\;\;\;\;\;\;\;\;\;\;\;\;+\sum_{j\leq q}^{}\left\{\underset{n\to \infty }{\lim}p\left(\left|R_{\mathrm{EYj}}^{}\right|>\varepsilon_{}^{*}\right)+\underset{n\to \infty }{\lim}p\left(\left|R_{\mathrm{VYj}}^{}\right|>\varepsilon_{}^{*}\right)\right\}\\ \leq 0\; \end{array}$$

which concludes that (A1) is true. Thus, we have completed the first part of proof of the Theorem A1, i.e., Equation (8) is a statistically consistent estimation for all the regression coefficients in Equation (6).

As for the second part of the proof, we will prove approximate unbiasedness of Equation (8); that is, we need to show

(A6) $$\underset{n\to \infty }{\lim}E\underset{j,k}{\max}\left|{\hat{\beta }}_{\mathrm{jk}}^{}-\beta_{\mathrm{jk}}^{}\right|=0.$$

where *E*(X) denotes the expectation of a random variable X. Observe that

(A7) $$\begin{array}{c} 0\leq \underset{k}{\max}\left|{\hat{\beta }}_{\mathrm{jk}}^{}-\beta_{\mathrm{jk}}^{}\right|\leq \sum_{l<p}^{}\left|{\hat{\beta }}_{\mathrm{jl}}^{}-\beta_{\mathrm{jl}}^{}\right|\left(1+\left|r_{l}^{*}\right|\right)+\sum_{s<p}^{}\underset{l}{\max}\left|\beta_{\mathrm{jl}}^{}\right|\left|{\hat{r}}_{s}^{}-r_{s}^{}\right|\\ \;\;\;\;\;\;\;\;\;\;\;\;\;\;\;\;\;\;\;\;\;\;\;\;\;\;\;\;+\sum_{t<p}^{}\underset{l}{\max}\left|\beta_{\mathrm{jl}}^{}\right|\left|R_{\mathrm{Ut}}^{}+R_{\mathrm{EXt}}^{}+R_{\mathrm{VXt}}^{}\right|+\left|R_{\mathrm{VYj}}^{}\right|\;+\left|R_{\mathrm{EYj}}^{}\right| \end{array}$$

If we can show that

(A8) $$\underset{n\to \infty }{\lim}E\left|{\hat{\beta }}_{\mathrm{jl}}^{}-\beta_{\mathrm{jl}}^{}\right|=\underset{n\to \infty }{\lim}E\left|{\hat{r}}_{s}^{}-r_{s}^{}\right|=0$$

and that

(A9) $$\underset{n\to \infty }{\lim}E\left|R_{\mathrm{Ut}}^{}+R_{\mathrm{EXt}}^{}+R_{\mathrm{VXt}}^{}\right|=\underset{n\to \infty }{\lim}E\left|R_{\mathrm{VYj}}^{}\right|=\underset{n\to \infty }{\lim}E\left|R_{\mathrm{EYj}}^{}\right|=0,$$

the proof of the Theorem A1 will be complete, i.e., (A6) is true, because, based on (A7)–(A9),

$$\begin{array}{l} \underset{n\to \infty }{\lim}E\underset{j,k}{\max}\left|{\hat{\beta }}_{\mathrm{jk}}^{}-\beta_{\mathrm{jk}}^{}\right|\leq \underset{n\to \infty }{\lim}\sum_{j\leq q}^{}\left\{\sum_{l<p}^{}\left(1+\left|r_{l}^{*}\right|\right)E\left|{\hat{\beta }}_{\mathrm{jl}}^{}-\beta_{\mathrm{jl}}^{}\right|+\underset{l}{\max}\left|\beta_{\mathrm{jl}}^{}\right|\sum_{s<p}^{}E\left|{\hat{r}}_{s}^{}-r_{s}^{}\right|\right.\\ \;\;\;\;\;\;\;\;\;\;\;\;\;\;\;\;\;\;\;\left.+\underset{l}{\max}\left|\beta_{\mathrm{jl}}^{}\right|\sum_{t<p}^{}E\left|R_{\mathrm{Ut}}^{}+R_{\mathrm{EXt}}^{}+R_{\mathrm{VXt}}^{}\right|+E\left|R_{\mathrm{VYj}}^{}\right|\;+E\left|R_{\mathrm{EYj}}^{}\right|\right\}\\ \;\;\;\;\;\;\;\;\;\;\;\;\leq \left(1+\underset{s}{\max}\left|r_{s}^{*}\right|\right)\sum_{j\leq q}^{}\left\{\sum_{l<p}^{}\underset{n\to \infty }{\lim}E\left|{\hat{\beta }}_{\mathrm{jl}}^{}-\beta_{\mathrm{jl}}^{}\right|+\underset{n\to \infty }{\lim}E\left|R_{\mathrm{VYj}}^{}\right|\;+\underset{n\to \infty }{\lim}E\left|R_{\mathrm{EYj}}^{}\right|\right\}\\ \;\;\;\;\;\;\;\;\;\;\;\;\;\;\;+q\underset{j,k}{\max}\left|\beta_{\mathrm{jk}}^{}\right|\left\{\sum_{s<p}^{}\underset{n\to \infty }{\lim}E\left|{\hat{r}}_{s}^{}-r_{s}^{}\right|+\sum_{t<p}^{}\underset{n\to \infty }{\lim}E\left|R_{\mathrm{Ut}}^{}+R_{\mathrm{EXt}}^{}+R_{\mathrm{VXt}}^{}\right|\right\}\\ \leq 0. \end{array}$$

The remaining part of the proof now is to show that (A8) and (A9) hold. We will first show that (A9) is true. According to the assumption of independent and identical sampling in design specifications D1, D3, and D5, and to Lemma-(iv) and the analogous arguments of proof of (A.6) in [17], there must exist a random variable *D_VY_*, which is greater than |*R_VYj_* | such that the expectation of *D_VY_* is finite (smaller than infinity). Following (A4) and the *theorem of dominated convergence* (see, for example, [54]), we have

$$\underset{n\to \infty }{\lim}E\left|R_{\mathrm{VYj}}^{}\right|=0.$$

Similarly, we have

$$\underset{n\to \infty }{\lim}E\left|R_{\mathrm{EYj}}^{}\right|=\underset{n\to \infty }{\lim}E\left|R_{\mathrm{Ut}}^{}\right|=\underset{n\to \infty }{\lim}E\left|R_{\mathrm{EXt}}^{}\right|=\underset{n\to \infty }{\lim}E\left|R_{\mathrm{VXt}}^{}\right|=0,$$

which concludes that (A9) holds. Analogously, we come to the conclusion that (A8) also holds via repeating similar arguments of proof for (A9).

As above, we have completed the proof of Theorem A1. □

## Appendix B

**Table A1:** The association parameters of the GAN inferred by AWTE from a DLBCL gene expression dataset (GSE60).

	p21	MYC	p53	BCL2	NFKB1	IRF4	Blimp1	AID	p27	ATR
**BCL6**	0.059	−0.267	0.158	**−0.686**	0.108	**−0.667**	−0.022	0.053	**−0.263**	−0.049
**SPIB**	−0.110	0.235	**−0.236**	0.093	0.018	**0.677**	0.026	**0.650 ***	−0.042	−0.122
**BACH2**	−0.108	−0.052	0.048	0.168	**−0.303**	**−0.255**	**−0.296**	**−0.563 ***	**0.132**	**0.158**
**IRF8**	−0.234	−0.233	−0.050	0.101	**−0.249**	**−0.329 ***	**−0.189**	−0.226	0.003	0.068
**OCT2**	−0.134	0.055	0.001	**0.361**	−0.113	**0.554**	0.163	**0.532**	−0.017	−0.005

This table is a representation of the GAN derived by AWTE for the 5 genes (*BCL6*, *SPIB*, *BACH2*, *IRF8*, and *OCT2*) that regulate 10 genes (*p21*, *MYC*, etc.) in the germinal center B cell, with the numerical values being the association parameters determined. Dotted boxes are twenty associations (network edges) reported in the literature; the 8 associations in addition to the 12 shown in Figure 1 of [41] are: SPIB-IRF4 [55], BACH2-BCL2 [47], OCT2-MYC [56], OCT2-BCL2 [57], OCT2-NFKB1 [56], OCT2-IRF4 [56], OCT2-Blimp1 [58], and OCT2-AID [59]. Those inferred by ROC curve analysis to yield the best AUC (Figure 5; 0.26 being the cut-off) are underlined. Those with *t*-test under the null hypothesis *β* = 0 being significant (*p*-value < 0.05) are boldfaced (see Table A2 below for the *p*-values). The three asterisked associations have support from mouse studies: SPIB-AID [44], BACH2-AID [45], and IRF8-IRF4 [46].

**Table A2:** Statistical test results (*p*-values) for the association parameters of the GAN inferred by AWTE from a DLBCL gene expression dataset (GSE60).

	p21	MYC	p53	BCL2	NFKB1	IRF4	Blimp1	AID	p27	ATR
**BCL6**	0.733	0.227	0.242	**1.0 × 10^−6^**	0.261	**1.4 × 10^−4^**	0.820	0.736	**0.003**	0.521
**SPIB**	0.467	0.222	**0.047**	0.427	0.828	**1.2 × 10^−5^**	0.759	**6.0 × 10^−6^**	0.575	0.069
**BACH2**	0.355	0.723	0.595	0.062	**5.9 × 10^−6^**	**0.027**	**8.9 × 10^−6^**	**4.4 × 10^−7^**	**0.024**	**0.002**
**IRF8**	0.096	0.192	0.644	0.351	**0.002**	**0.018**	**0.015**	0.078	0.971	0.268
**OCT2**	0.424	0.795	0.997	**0.006**	0.224	**0.001**	0.079	**6.7 × 10^−4^**	0.834	0.944

Those with *t*-test under the null hypothesis *β* = 0 being significant (*p*-value < 0.05) are boldfaced.

**Table A3:** The association parameters of the GAN inferred by LSE (using GSE60).

	p21	MYC	p53	BCL2	NFKB1	IRF4	Blimp1	AID	p27	ATR
**BCL6**	0.041	−0.181	0.060	**−0.586**	0.099	**−0.372**	−0.017	**0.299**	**−0.286**	−0.048
**SPIB**	0.221	**0.711**	**−0.311**	0.039	−0.116	**0.509**	−0.107	**0.615**	**0.211**	0.099
**BACH2**	**−0.171**	−0.040	0.055	0.113	**−0.245**	−0.078	**−0.298**	**−0.358**	**0.116**	**0.134**
**IRF8**	−0.079	**−0.364**	0.057	0.163	−0.094	**−0.371**	−0.045	**−0.225**	0.002	0.047
**OCT2**	**−0.443**	**−0.689**	**0.323**	**0.259**	0.025	**0.205**	0.250	0.119	**−0.260**	**−0.206**

Those with *t*-test under the null hypothesis *β* = 0 being significant (*p*-value < 0.05) are boldfaced.

**Table A4:** The association parameters of the GAN inferred by RRE (using GSE60).

	p21	MYC	p53	BCL2	NFKB1	IRF4	Blimp1	AID	p27	ATR
**BCL6**	0.029	−0.170	0.051	**−0.536**	0.071	**−0.321**	−0.018	**0.285**	**−0.267**	−0.039
**SPIB**	**0.172**	**0.646**	**−0.248**	0.034	−0.077	**0.431**	−0.076	**0.581**	**0.186**	0.069
**BACH2**	**−0.187**	−0.072	0.081	0.110	**−0.207**	−0.104	**−0.266**	**−0.359**	**0.100**	**0.108**
**IRF8**	−0.064	**−0.330**	0.036	0.152	−0.094	**−0.310**	−0.053	**−0.199**	0.008	0.049
**OCT2**	**−0.390**	**−0.654**	**0.282**	**0.243**	0.006	**0.194**	**0.218**	0.120	**−0.245**	**−0.174**

Those with *t*-test under the null hypothesis *β* = 0 being significant (*p*-value < 0.05) are boldfaced.

**Table A5:** The association parameters of the GAN inferred by LASSO (using GSE60).

	p21	MYC	p53	BCL2	NFKB1	IRF4	Blimp1	AID	p27	ATR
**BCL6**	0.022	−0.122	0.039	**−0.514**	0.067	**−0.307**	−0.010	**0.228**	**−0.236**	−0.032
**SPIB**	0.156	**0.541**	**−0.242**	0.024	−0.079	**0.440**	−0.074	**0.544**	**0.157**	0.073
**BACH2**	−0.166	−0.070	0.062	0.094	**−0.208**	−0.073	**−0.254**	**−0.316**	0.083	**0.109**
**IRF8**	−0.047	**−0.266**	0.035	0.137	−0.077	**−0.306**	−0.035	−0.155	0.001	0.038
**OCT2**	**−0.381**	**−0.600**	**0.281**	**0.236**	0.014	**0.163**	**0.197**	0.084	**−0.218**	**−0.176**

Those with *t*-test under the null hypothesis *β* = 0 being significant (*p*-value < 0.05) are boldfaced.

**Table A6:** GAN method comparisons among four different distribution-free regression approaches.

GAN Method Comparison *	Close Form for Estimating Equation	Collinear Impact Adjustment	Noise/Heterogeneity Tolerating	Large Scale Predictor Gene Selection
LSE	Yes	No	No	No
RRE	Yes	Yes	No	No
LASSO	No	Yes	No	Yes
AWTE	Yes	Yes	Yes	No

* Using regression methods for comparison of GAN construction in the case of non-time series gene expression data. Gaussian graphical models and Bayesian models, though not distribution-free, generally have similar patterns as LASSO.
